# Algorithm for identifying and separating beats from arterial pulse records

**DOI:** 10.1186/1475-925X-4-48

**Published:** 2005-08-11

**Authors:** Ernesto F Treo, Myriam C Herrera, Max E Valentinuzzi

**Affiliations:** 1 Departamento de Bioingeniería, *Instituto Superior de Investigaciones Biológicas *(INSIBIO), Consejo Nacional de Investigaciones Científicas y Técnicas (CONICET), Universidad Nacional de Tucumán (UNT), CC327, 4000, Tucumán, Argentina; 2 Departamento de Bioingeniería, *Instituto Superior de Investigaciones Biológicas *(INSIBIO), Consejo Nacional de Investigaciones Científicas y Técnicas (CONICET), Universidad Nacional de Tucumán (UNT), CC327, 4000, Tucumán, Argentina. Also with Facultad de Ciencias Exactas y Tecnología (FACET), Universidad Nacional de Tucumán (UNT), Av. Independencia 1800, 4000, Tucumán, Argentina; 3 Departamento de Bioingeniería, *Instituto Superior de Investigaciones Biológicas *(INSIBIO), Consejo Nacional de Investigaciones Científicas y Técnicas (CONICET), Universidad Nacional de Tucumán (UNT), CC327, 4000, Tucumán, Argentina

**Keywords:** second derivative beat detection, limb impedance plethysmography, patient screening, preventive medicine

## Abstract

**Background:**

This project was designed as an epidemiological aid-selecting tool for a small country health center with the general objective of screening out possible coronary patients. Peripheral artery function can be non-invasively evaluated by impedance plethysmography. Changes in these vessels appear as good predictors of future coronary behavior. Impedance plethysmography detects volume variations after simple occlusive maneuvers that may show indicative modifications in arterial/venous responses. Averaging of a series of pulses is needed and this, in turn, requires proper determination of the beginning and end of each beat. Thus, the objective here is to describe an algorithm to identify and separate out beats from a plethysmographic record. A secondary objective was to compare the output given by human operators against the algorithm.

**Methods:**

The identification algorithm detected the beat's onset and end on the basis of the maximum rising phase, the choice of possible ventricular systolic starting points considering cardiac frequency, and the adjustment of some tolerance values to optimize the behavior. Out of 800 patients in the study, 40 occlusive records (supradiastolic- subsystolic) were randomly selected without any preliminary diagnosis. Radial impedance plethysmographic pulse and standard ECG were recorded digitizing and storing the data. Cardiac frequency was estimated with the Power Density Function and, thereafter, the signal was derived twice, followed by binarization of the first derivative and rectification of the second derivative. The product of the two latter results led to a weighing signal from which the cycles' onsets and ends were established. Weighed and frequency filters are needed along with the pre-establishment of their respective tolerances. Out of the 40 records, 30 seconds strands were randomly chosen to be analyzed by the algorithm and by two operators. Sensitivity and accuracy were calculated by means of the true/false and positive/negative criteria. Synchronization ability was measured through the coefficient of variation and the median value of correlation for each patient. These parameters were assessed by means of Friedman's ANOVA and Kendall Concordance test.

**Results:**

Sensitivity was 97% and 91% for the two operators, respectively, while accuracy was cero for both of them. The synchronism variability analysis was significant (*p *< 0.01) for the two statistics, showing that the algorithm produced the best result.

**Conclusion:**

The proposed algorithm showed good performance as expressed by its high sensitivity. The correlation analysis demonstrated that, from the synchronism point of view, the algorithm performed the best detection. Patients with marked arrhythmic processes are not good candidates for this kind of analysis. At most, they would be singled out by the algorithm and, thereafter, to be checked by an operator.

## Background

Outpatients coming daily for consultation to a general public hospital are often preventively checked for signs suggestive of infectious, cardiovascular and/or any other endemic disease. The positive detected fraction is derived for further confirmatory study, which may lead to eventual treatment. Within such concept, this project was specifically designed as an epidemiological aid-selecting tool for a small country health center serving a large rural area (see Acknowledgments). Essential requirements were low cost and simplicity. The general objective was to screen out possible coronary patients.

Peripheral artery function can be non-invasively evaluated by impedance plethysmography, either in lower or upper limbs [1]. Changes in these vessels appear as good predictors of future coronary behavior [2,3]. Basically, impedance plethysmography detects volume variations due to the pulsating blood flow that, after simple mechanical occlusive maneuvers, may show indicative modifications in arterial/venous responses [4,5].

Pulse plethysmographic analysis, based on variations of its amplitude or waveform [6], requires the averaging of several beats.

There are specific algorithms for the detection of the dicrotic notch [7]; some papers make a beat-to-beat analysis of the arterial pressure [8-11]. Commercial equipment (like Complior ^®^SP, Artech Medical,  y SphygmoCor ^® ^Vx, Atcor Medical, ) carry out the above mentioned type of plethysmographic signal analysis. Schroeder *et al *[12], by means of MATLAB, developed a cardiovascular package (named HEART), which permits beat identification using two sequential processes (one of coarse approximation and a second one of fine adjustment). Unfortunately, none of these procedures offer detailed descriptions.

Besides, several algorithms have been developed to detect electrocardiographic beats, by and large based on the recognition of the QRS complex [13]. However, our design has been thought to operate independently of the ECG signal; for these reasons they are not applicable in this case.

Thus, the objective here is to describe an algorithm to identify and separate out beats from a plethysmographic record during an occlusive maneuver. As a secondary objective, we intended to compare the output given by human operators (trained and not trained) against the algorithm. The method herein proposed is potentially applicable to other cardiac signals.

## Methods

### Outline

An occluding cuff produces a limb short ischemia. Basal and post-occlusion plethysmographic arterial pulse records are compared searching for either amplitude and/or waveform modifications or both. Since the possible change in a single beat record does not supply enough information, valid results call for averaging of a series of pulses and this, in turn, requires proper determination of the beginning and end of each beat. In other words, good beats must be identified and singled out discarding abnormal pulses.

**Figure 1:**
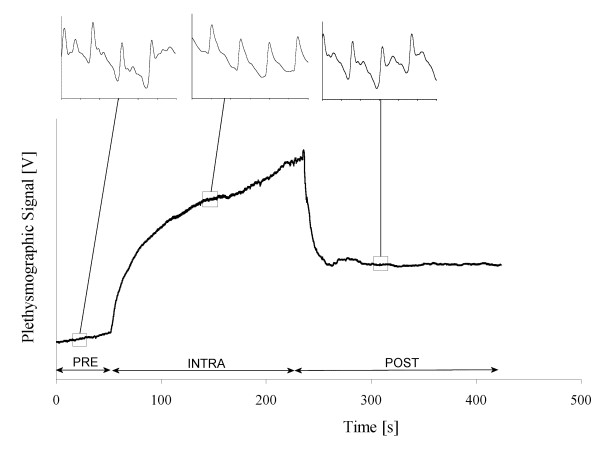
Typical dc plethysmographic record of an occlusive maneuver. Insets show expanded pulses during each stage; differences are evident. An arbitrary zero time is also showed from which the pre-inflation time was measured (PRE); thereafter, the intra-occlusion (INTRA) period followed (bounded by the beginning and end of inflation), until post-occlusion was reached (POST).

Each applied maneuver consists of a supradiastolic and subsystolic occlusion; thus, it permits inflow of blood while stops outflow, which leads to limb volume increase. A typical record is showed in Figure 1. The pulse identification algorithm detected the beat's onset and end on the basis of the maximum rising phase, the choice of possible ventricular systolic starting points considering cardiac frequency, and the adjustment of some tolerance values to optimize the behavior.

### Patient population

From the daily inflow of hospital outpatients, we obtained 800 records out of which 40 supradiastolic-subsystolic occlusive ones were randomly selected without any preliminary diagnosis. All patients accepted and signed the informed consent. The attending physician, including the measurements leading to the quantitative data mentioned above, carried out routine clinical interrogation. Blood pressure was obtained with the oscillometric method using the contra-lateral arm to that where the test was to be performed. Patients rested for at least 5 min in the supine position prior to the test.

### Impedance Plethysmography and Recording System

Radial pulse was picked up with two metallic electrodes (ECG standard type) placed over the forearm artery line, 2 cm below the ante-cubital fold, and 5 to 10 cm apart. The forearm was always at the left atrial level. Besides, a simultaneous standard ECG was obtained. Impedance was obtained with a custom-made laboratory apparatus [14].

Digital acquisition (sampling frequency *sf *= 200 Hz, at 16 bits) was carried out using a commercial system (BIOPAC System Inc, AcqKnowledge II for MP100WSW). Each occlusive maneuver record included basal plethysmographic pulses (PRE), a period of 2 to 3 minutes of occlusive cuff inflation (INTRA), and a post-occlusive (POST) after release; the overall duration was always in the order of 5–6 min (Figure 1).

### Algorithm

**Figure 2:**
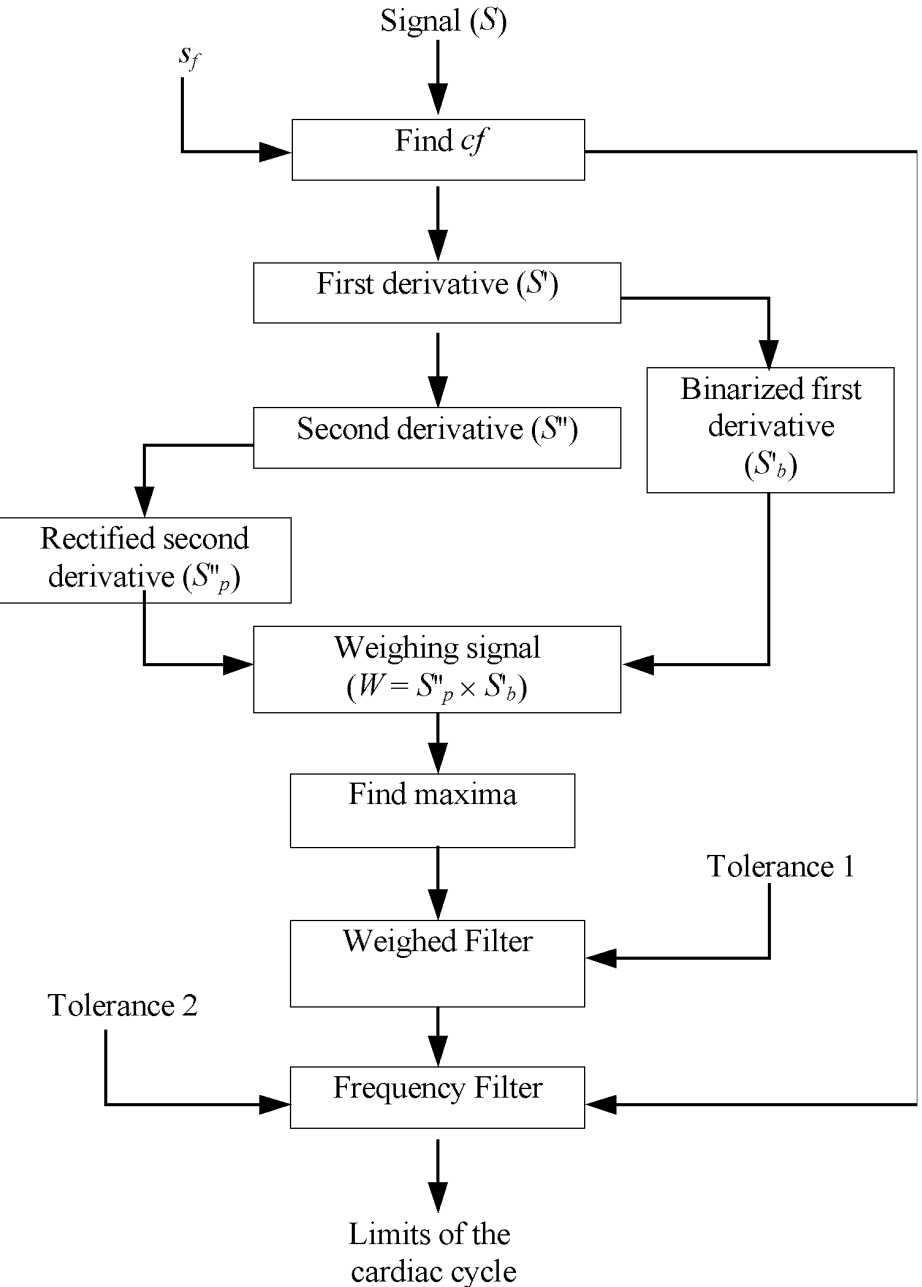
Flow Diagram of the Algorithm.

Figure 2 summarizes the sequential steps the recorded signal *S *went through. The first step is the detection of the average cardiac frequency *fc*, which is expected to be within the 0.5 Hz – 2.5 Hz range, and is divided in two stages: First, the signal goes through a band-pass FIR (Finite Impulsive Response) filter, with cut-off frequencies of 0.8 Hz and 2.8 Hz, and attenuation at least of -50dB at 0.25 Hz and 3.25 Hz. Thereafter, the Power Spectral Density function (PSD, an averaging variant of the Fast Fourier Transform), is applied to find *fc*. The first minute of data acquisition corresponds to the basal or pre-occlusion stage (Fig. 1). This period is divided into overlapping sections; each is linearly detrended, then windowed with a Hanning function (4,096 samples) and, thereafter, zero-padded to a length of 8,192 samples. The magnitude squared of the Fast Fourier Transform of each section is averaged to form *P_xx_*, the Spectral Density Function. Each section overlaps with the previous 2000 samples. The maximum value for the first harmonic component corresponds to the cardiac frequency *fc *(see Signal Processing Toolbox for Use with MATLAB, The MathWorks, Inc, Natick, MA).

The original signal is derived twice applying the well-known iterative series of subtractions [15], that is,

*S*'(*n*) = *S*(*n *+ 1) - *S*(*n*)

**Figure 3:**
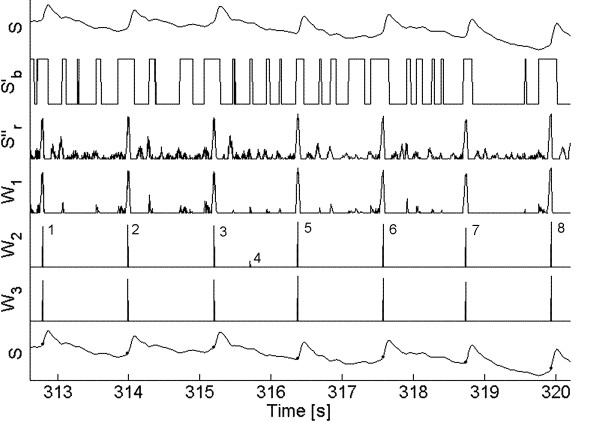
Different stages while processing a typical signal. *S *is the original signal; *S*'_*b *_is the binarized first derivative; *S*''_*r *_is the rectified second derivative; *W*_1 _is the weighing signal showing all spikes detected by marks; *W*_2 _is the same weighing signal, with only those spikes preserved by the weighed filter. *W*_3 _is the same weighing signal with only those spikes preserved by the frequency filter. The bottom trace represents again *S*, with all onsets and ends well identified. Tolerance values were *Tol*_1 _= 0.4 and *Tol*_2 _= 0.2.

*S*"(*n*) = *S*'(*n *+ 1) - *S*'(*n*)     [1]

where *S*'(*n*) and *S*"(*n*) stand, respectively, for the first and second derivative and *n *represents the sample number. Figure 3, upper trace, shows 6 beats of a typical plethysmographic record. Thereafter, both signals are processed binarizing the first derivative and rectifying the second derivative. Binarization means to replace 1's for positive values and 0's for negative ones. The process of rectification leaves only the positive excursions. Clearly, the product of the binarized and rectified signals is a trace showing large peaks and some significantly lower ones in between, which is called the weighing signal *W*_1_. When the latter product is compared with the original *S *signal, one can easily see that the large peaks are obviously coincident with true good beats while the small spikes correspond to other changes (not pulses) in that signal. A spike detection routine based on the first derivative sign change identifies all maxima, including both the large and the small spikes; thus, filtering is required to remove the small spikes and preserve the large ones.

### Weighed Filter

The sequence of maximum spikes after multiplication (Figs. 2 and 3) can be used as a filtering criterion to separate out the true beats. Whenever the interval between two pulses is much smaller than the cardiac period (for example, less than one half), it can be assumed that the two spikes are too close together and cannot represent a beginning (and ending) of a whole cycle. Consequently, one must be removed.

Since, by and large, the beginning of a cycle corresponds to a steep rise time, the second derivative has more weight, and the multiplication result clearly indicates to retain that particular spike (Fig. 3, trace *W*_2_).

Mathematically, this is treated as

*t*(*S*_i+1_) - *t*(*S*_i_) <*Tol*_1 _× *T*_*c *_    [2]

where *t*(*S*_i+1_) and *t*(*S*_i_) are, respectively, the time of appearance of spikes *i*+1 and *i *in signal *W*_1_, *Tol*_1 _is a preset tolerance value and *T*_*c *_= 1/*fc *stands for the cardiac period expressed in seconds. Each pair of adjacent spikes is analyzed and, if the comparison result is true, the smallest is removed and the new pair of contiguous spikes is now chosen. If the result is false, *i *is incremented. The process repeats until no true result is obtained. Adjusting the tolerance value *Tol*_1_, the number of removed spikes can be increased or decreased.

### Frequency Filter

Once all small spikes have been removed, the remaining spikes must be analyzed to check if they correspond to the beginning and end of a cycle. Figure 3 (fifth trace *W*_2_) shows the peaks remaining after the weighed filter. Spike 4 is a misdetection that must be removed. A second filter matches pairs of peaks (not necessarily consecutive) checking whether they correspond to a beat limits or not; the distance between them should be fixed between *T*_*c *_± *Tol*_2_, where the latter is a second tolerance value, generally chosen close to 20%.

Starting from the first detected spike 1 at time *t *(Fig. 3, fifth trace), and assuming it corresponds to the beginning of a cycle, a second spike should be located within the interval *t *+ *T*_*c *_± 20%. If this second spike exists, the time corresponding to both spikes is stored as the limits of a cardiac cycle. In fact, this second spike exists in Figure 3, marked as 2. The process is repeated starting now from 2 and so on. Now, let us consider that the first spike detected was 4. When searching its partner spike ahead, the algorithm will not find it because 5 and 6 are, respectively, too close and too far from 4. In this case, 4 is discarded and the process continues to the next one.

Cardiac period has been assumed constant up to now, however, it is known to be modulated by the respiratory heart rate response. To have a better estimation of *T*_*c*_, each time two spikes are found to be (*T*_*c *_± *Tol*_2_) seconds apart, their difference is used to update a new value of *T*_*c *_to be applied in the following calculations.

### Statistics

For each of the 40 patients, a 30 s trace was chosen at random, which was analyzed by two operators. One of them (operator 1) was trained and familiar with the procedure and another (operator 2) without any previous training. Both operators received the same instructions regarding the analysis to be performed. Each operator marked manually the beginning and end of each beat as the 30 s sample was presented on the monitor. The selection criterion was to identify that point previous to the rapid rising of the ejective period, not necessarily coincident with the previous minimum. In this way, there were two marks that clearly bounded each positive cycle. When the beat limits were not clearly defined or the signal was lost due to circuit saturation, the portion between the last observed beat and the following beginning was classified as negative (i.e., rejected). Thereafter, the algorithm was applied and coincidences with the operators' results were searched.

**Figure 4:**
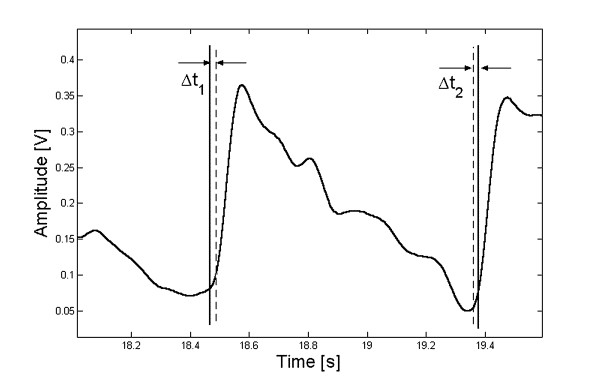
A single beat indicating the maximum error admitted for the statistical analysis.

Since an exact coincidence is almost nil, we adopted a threshold level to specify the maximum difference to be accepted between the limits marked by the operators and the algorithm. A program was developed to determine the beginning and ending points closest to those selected by the operator (Fig. 4). When the addition of the differences (Δt_1 _+ Δt_2_) was lower than 10% of the cardiac period, the beat was considered as *true positive *(*tp*). If the difference was larger, the beat was classified as *false negative *(*fn*). However, when the operator could not bound the beat there is an undetermined signal length, which must not be processed. If coincidently the algorithm did not classify any beat within that same length, the selection is marked as *true negative *(*tn*). Instead, if the algorithm picked up at least one beat, the selection is considered as *false positive *(*fp*).

*Sensitivity *of the procedure was defined as the percentage of beats correctly selected by the algorithm with respect to the total number of beats marked as true by the operator, that is,

*s *[%] = *tp /*(*tp *+ *fn*) × 100     [3]

*Accuracy*, instead, was defined as the total number of sections correctly rejected by the algorithm with respect to the total number of sections discarded by the operator,

*a *[%] = *tn */(*tn *+ *fp*) × 100     [4]

Bounding of the beats is also important for the correct synchronization of the averaging procedure. Thus, those beats correctly classified by all three methods (operators 1 and 2 and the algorithm) were selected to compare the synchronization ability. For that matter, the time between the beginning and the first maximum coincident with ventricular ejection was measured for each beat. This time was, of course, different for the operators and the algorithm, each with a specific coefficient of variation. The latter was taken as the statistical estimator.

Moreover, for each patient a correlation analysis was carried out between all possible combinations of the *tp *beats. For each pair of beats a correlation factor was obtained, thus producing a non-normal distribution when all combinations are considered, which is usually characterized by the median value. In the end, we obtained three of these values for each patient according to the classification methods (two operators and algorithm).

The coefficient of variation (also with a non-Gaussian distribution) and the median should be analyzed by non-parametric techniques. In our case, we used Friedman's ANOVA and Kendall Concordance.

## Results

**Figure 5:**
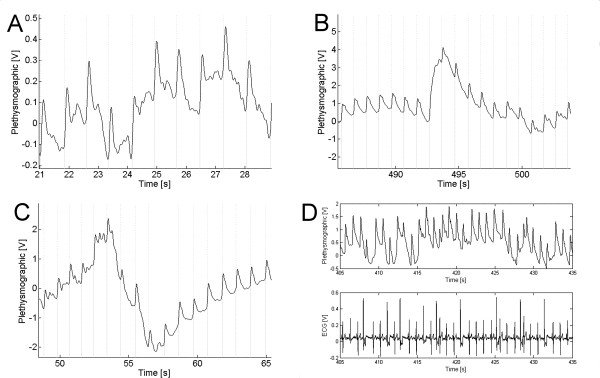
Several signals and the results of the algorithmic process. Vertical lines mark the beginning (dotted) and end (dashed) of each cycle. (A) Patient with Parkinson disease; (B) Deflation of the cuff; (C) Inflation of the cuff. The two latter in the same patient. Section D shows the ECG and the plethysmographic signal from a patient with cardiac arrhythmia.

Figure 5 shows three examples of signals and their bounding obtained by the algorithm. Its last section D belongs to an arrhythmic patient.

The sensitivity was 97% and 91% for operators 1 and 2, respectively. Patients with low sensitivity were retrospectively analyzed. Only one patient produced a low sensitivity according to the criteria of both operators; after careful analysis, we found large heart rate variability mainly due to ectopic beats (Fig. 5D, Table 1).

**Table 1:** Results of the statistical analysis for both operators.

		Operator 1			Operator 2	
				
		POSITIVE	NEGATIVE		POSITIVE	NEGATIVE
Algorithm	POSITIVE	1403 (*tp*)	5 (*fp*)	POSITIVE	1321 (*tp*)	7 (*fp*)
	NEGATIVE	44 (*fn*)	0 (*tn*)	NEGATIVE	124 (*fn*)	0 (*tn*)

Accuracy for both operators was 0 because traces marked as negative were somehow classified by the algorithm, thus, producing *fp *beats.

**Figure 6:**
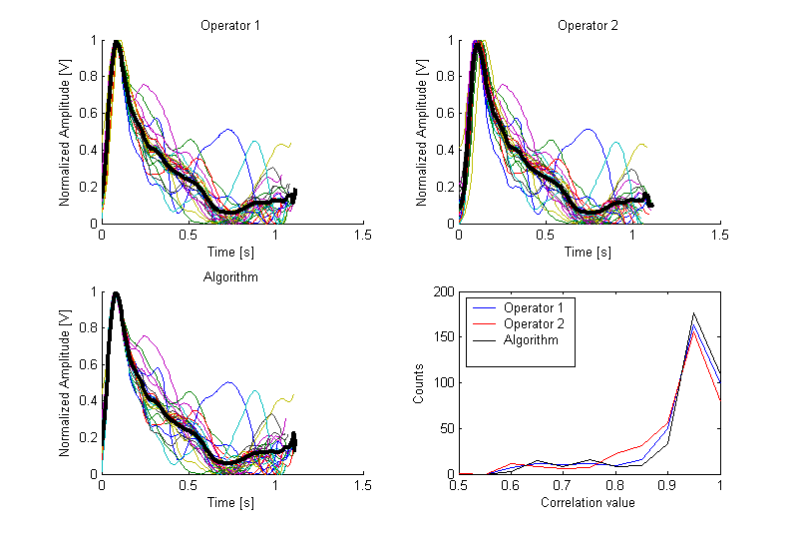
Superimposed separated beats (thin colored lines) and one averaged beat (thick black line), from a particular patient, as they were singled out by (A) Operator 1, (B) operator 2 and (C) the algorithm. Section (D) shows the histogram of correlation values for all beats.

The synchronism variability analysis gave off significant values *p *(*p < 0.01*) for the two statistics. However, Kendall coefficients were 0.62 and 0.17, respectively, for the median correlation and the coefficient of variation. Such results suggest that synchronism is not the same for the three separation criteria. Figure 6 shows the typical overlapping of several beats along with their averaged result. This represents a good way of visualizing what could be the pattern of, say, a normal beat, which could serve as a comparison reference. The same beats were separated according to the criteria of the two operators and the algorithm. The right lower graph shows a histogram with the correlation coefficients of all beats. Very few produced low values while most of them are grouped rather close to 1; as expected, the algorithm produced the maximum of the three.

The detection routine for the average cardiac frequency was a critical factor in the analysis of the algorithm; any failure in it can produce an error that would propagate to any subsequent processing. Thus, this parameter was checked by visual inspection of the 40 signals and their frequency spectra.

## Discussion

This algorithm allows the averaging of non-invasively obtained arterial pulses for the evaluation of the vascular response to peripheral occlusive maneuvers employing only the plethysmographic signal. The ECG served as monitor of cardiac activity and was used to help the operators in their task. Since the algorithm was designed thinking of a possible commercial equipment based only on the plethysmographic signal, the ECG, cannot be included in the analysis.

**Figure 7:**
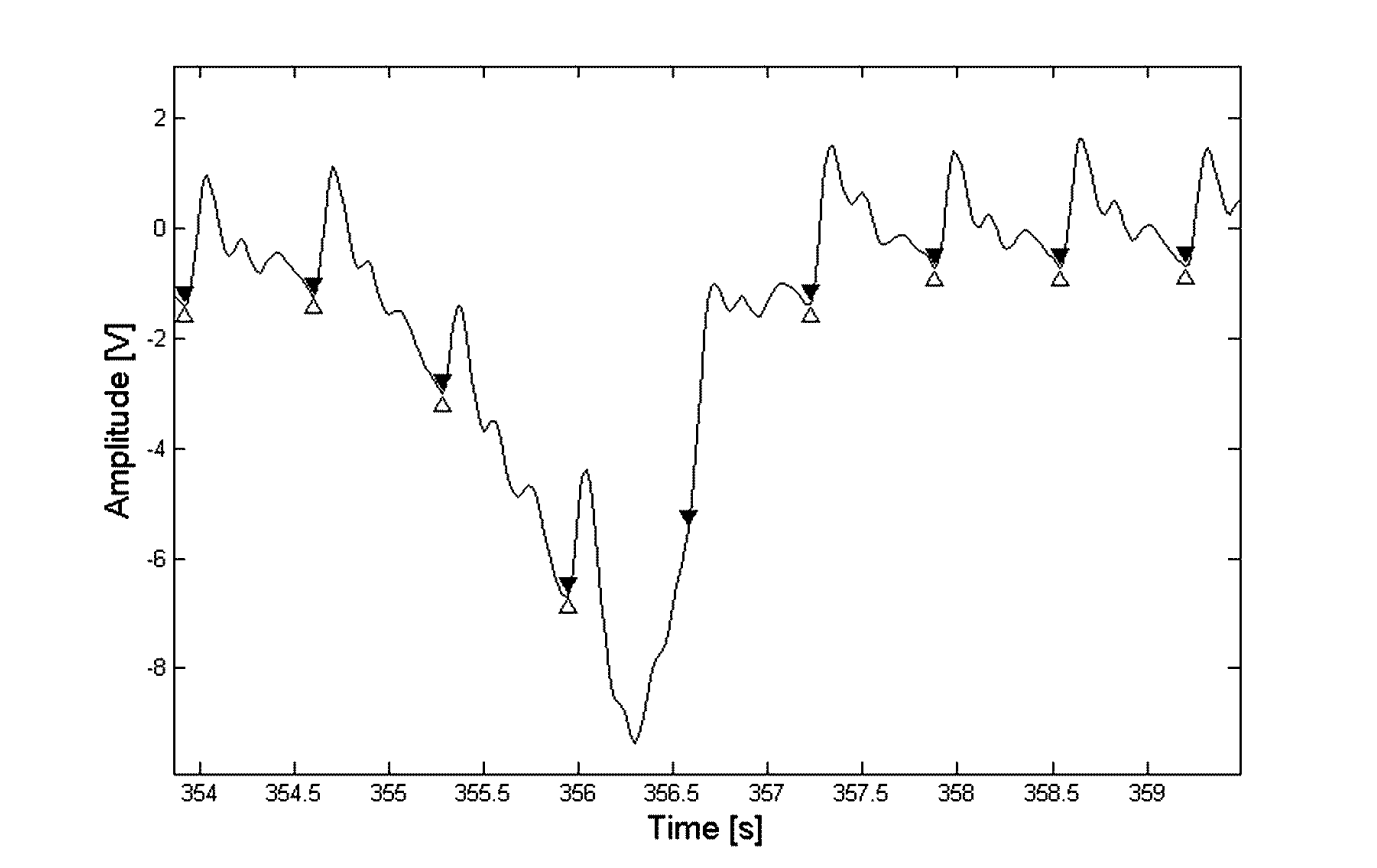
Typical case of a beat falsely classified as positive by the algorithm. Δ are the points selected by the operator and ▼ are those detected by the algorithm.

The algorithm sensitivity depends on the operator and it was always higher than 90%. However, accuracy was always cero. None of the sections marked as negative by any operator was correctly rejected by the algorithm. Analysis of the *fp *beats showed that the portions discarded by the operators are, by and large, sections with large drifts mainly due to cuff inflation or by movement artifacts. An important problem lies on the fact that the algorithm, many times, picks up beats that are placed in the course of a drift. An operator would never select such a beat, as exemplified in Figure 7. The possible utility of such *fp *beats perhaps ought to be studied because they might still contain clinical information.

The algorithm is based on the analysis of the maximum systolic slope discarding pulses not consistently separated out from their respective previous beats or when the derivative value is too low. Usually, beat separation in blood pressure records is obtained by the minimum value previous to the dicrotic notch. Noise, however, may perturb this kind of determination. When pulses selected by this criterion are overlapped for their averaging, the systolic maximum does not temporally coincide in all beats and a small shift, unpredictable and unknown, shows up. Low amplitude noise, when present, tends to interfere with the temporal location of the valve opening point. The maximum second derivative criterion does not really represent valve opening but rather represents maximum rise during systole. In most of the patients, in our experience, the latter reference showed better periodicity and seems to be a better time reference when averaging is required. The second event seems to be less sensitive to interferences because signal growth during systole is larger than noise changes. This observation was supported by the variability analysis. Kendall coefficient indicates that correlation is a reliable statistic and its variability is similar in the three methods.

The algorithm, due to its philosophy of design, does not have to identify all beats, so conferring to it a practical characteristic, i.e., rather frequently, due to patient's movements, the system's electronics may saturate. In such case, the algorithm disregards the piece resuming the search after signal recovery. However, the two filtering criteria are based on the cardiac frequency and, when the latter is too variable (for example, due to arrhythmias) the sensitivity falls drastically (figure 5D).

The average cardiac frequency is not affected during the occlusive maneuvers [16,17], and for patients without rhythm alterations, a mean cardiac frequency assumptions appears as reasonable.

Tolerances, in turn, are useful to modify the algorithm's performance according with the prevalent conditions (noise, drift, saturation). However, sensitivity higher than 90% is enough when the recording time is long (say, 5–6 min or more). The tolerance values suggested here produced in our opinion the best results.

Interested investigators are encouraged to request the algorithm in order to test it using signals obtained from other sources. These authors would be happy to make it available.

## Conclusion

The proposed algorithm showed good performance as expressed by its high sensitivity. The correlation analysis demonstrated that, from the synchronism point of view, the algorithm performed the best detection. Patients with marked arrhythmic processes are not good candidates for this kind of analysis. At most, these patients would be singled out by the algorithm to be checked by an operator.

## Authors' contributions

These authors contributed equally to this work; the former developed the basic idea of the algorithm while the latter actually obtained the records in the hospital environment. The corresponding last author gave orientation, revised the data, and wrote the paper in its different stages. Design of the protocol was a team collaborative task.
